# Thromboembolic complications in children with COVID-19 and MIS-C: A narrative review

**DOI:** 10.3389/fped.2022.944743

**Published:** 2022-08-11

**Authors:** Sandra Trapani, Chiara Rubino, Donatella Lasagni, Francesco Pegoraro, Massimo Resti, Gabriele Simonini, Giuseppe Indolfi

**Affiliations:** ^1^Department of Health Sciences, Meyer Children’s University Hospital, University of Florence, Florence, Italy; ^2^Pediatric Unit, Meyer Children’s University Hospital, Florence, Italy; ^3^Rheumatology Unit, Meyer Children’s University Hospital, University of Florence, Florence, Italy; ^4^Department of NEUROFARBA, Meyer Children’s University Hospital, University of Florence, Florence, Italy

**Keywords:** thrombosis, children, COVID-19, MIS-C, stroke, embolism

## Abstract

COVID-19 and multisystem inflammatory syndrome in children (MIS-C) have been associated with a higher incidence of hypercoagulability and thromboembolic events (TEs), even in children, leading to relevant morbidity, and mortality. However, our understanding of such complications in childhood is limited. To better understand the incidence, clinical manifestations, risk factors, and management of COVID-19 and MIS-C-related TEs in children, a review of the current literature and a brief update on pathophysiology are given. Sixty-two studies, describing 138 patients with TEs associated with COVID-19 or MIS-C, were included. The overall number of TEs was 157, as 16 patients developed multiple TEs: venous TEs represented the majority (54%), followed by arterial thrombosis (38%, mainly represented by arterial ischemic stroke-AIS), and intracardiac thrombosis (ICT) (8%). Within the venous TEs group, pulmonary embolism (PE) was the most frequent, followed by deep venous thrombosis, central venous sinus thrombosis, and splanchnic venous thrombosis. Notably, 10 patients had multiple types of venous TEs, and four had both venous and arterial thrombosis including a newborn. Most of them (79 cases,57%) had at least one predisposing condition, being obesity the most frequent (21%), especially in patients with PE, followed by malignancy (9%). In 35% of cases, no data about the outcome were available About one-third of cases recovered, 12% improved at discharge or follow-up, and 6% had persistent neurological *sequelae.* The mortality rate was 12%, with death due to comorbidities in most cases. Most fatalities occurred in patients with arterial thrombosis. Pediatricians should be aware of this life-threatening possibility facing children with SARS-CoV-2 infection or its multisystemic inflammatory complication, who abruptly develop neurological or respiratory impairment. A prompt intensive care is essential to avoid severe sequelae or even exitus.

## Introduction

Despite the severe acute respiratory syndrome coronavirus 2 (SARS-CoV-2) infection primarily causing a respiratory illness, it has been associated with hypercoagulability and thromboembolic events (TEs). TEs are frequent in hospitalized adults with severe novel coronavirus disease 2019 (COVID-19), resulting in high rates of disability and mortality (1, 2). However, although much is known about TEs in adults with COVID-19, data in children are limited. The overall prevalence of thrombosis in children with COVID-19 is markedly lower than in adults, which has limited our understanding of the disease in this age group (3). In addition, children and adolescents are uniquely affected by a post-infectious hyperinflammatory syndrome after SARS-CoV-2 infection, named multisystem inflammatory syndrome in children (MIS-C) (4). The risk of thrombosis is increased in patients with MIS-C and, especially, in those who develop severe ventricular dysfunction or coronary artery aneurysms (5, 6). The thromboembolic manifestations during COVID-19 and MIS-C, occur not only in venous districts including deep veins thrombosis (DVT), central venous sinuses (cerebral venous sinus thrombosis, CVST), pulmonary embolism (PE), and splanchnic veins thrombosis (SVT), but also in cerebral arteries, causing acute ischemic stroke (AIS), and, more rarely, in the coronaries or peripheral systemic arteries. Intracardiac thrombosis (ICT) has also been exceptionally described (5, 7). TEs seem to be more common in patients with pre-existing risk factors for thrombosis, such as cancer, obesity, chronic diseases, and in those with a central venous line (CVL). TEs occur more frequently in older children and adolescents, but they have been described at all ages, even in a preterm infant with a severe form of SARS-CoV-2 infection complicated by CVST (8). To better understand the incidence, clinical manifestations, risk factors, and management of SARS-CoV-2-related TEs in children, we extensively reviewed the current literature, including original articles, observational studies, case series, and case reports in the last 2 years. A brief update on pathophysiology is also given.

## Materials and methods

We used Embase^®^, MEDLINE^®^, and MEDLINE^®^-In Process for English-language studies published from January 2020 through June 2022. PubMed databases were searched combining the keywords “COVID-19” OR “SARS-CoV-2” OR “coronavirus” AND “MIS-C” OR “PIMS” AND “thrombosis” OR “thromboembolism” OR “stroke” OR “thromboembolic” OR “pulmonary embolism” AND any of the following: “child” OR “children” OR “pediatric” OR “infant” OR “adolescent.” Titles and abstracts in English were evaluated for eligibility. References of identified relevant articles were reviewed and papers from these sources were also included. Thereafter, a further selection was made including those articles in which one or more clinical data concerning patients with TEs could be obtained. The selected articles were reviewed by two independent authors (ST and CR) and judged on their relevant contribution to the study subject. Subsequently, according to the type of thrombosis, all TEs were categorized into arterial thrombosis (including the AIS, coronary, and peripheral artery), venous thromboembolisms (VTEs) (including CVSTs, DVTs, SVTs, and PE), and ICT. Epidemiological and clinical findings, laboratory values, data on treatment, and outcome were reported. TEs were further distinguished as occurring in children with COVID-19, when the patient had positive real-time polymerase chain reaction or antigen SARS-CoV-2 tests on nasal or nasopharyngeal swabs, or in patients with MIS-C, defined according to the US Center for Disease Control and Prevention (4).

## Results

**FIGURE 1:**
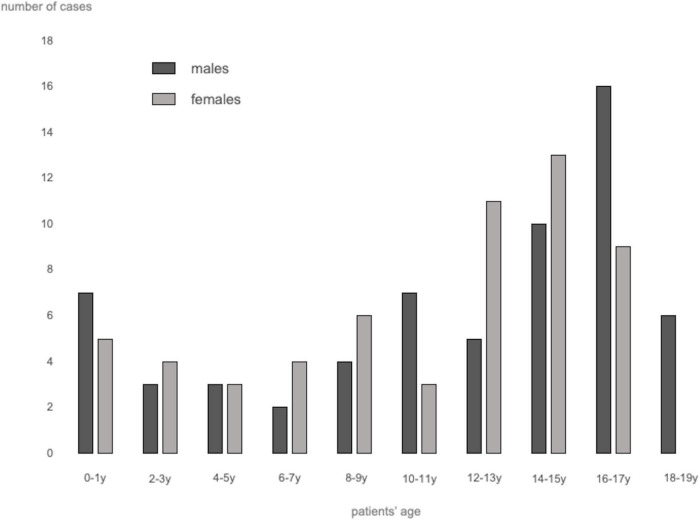
Distribution of thrombotic events by age and sex.

**FIGURE 2:**
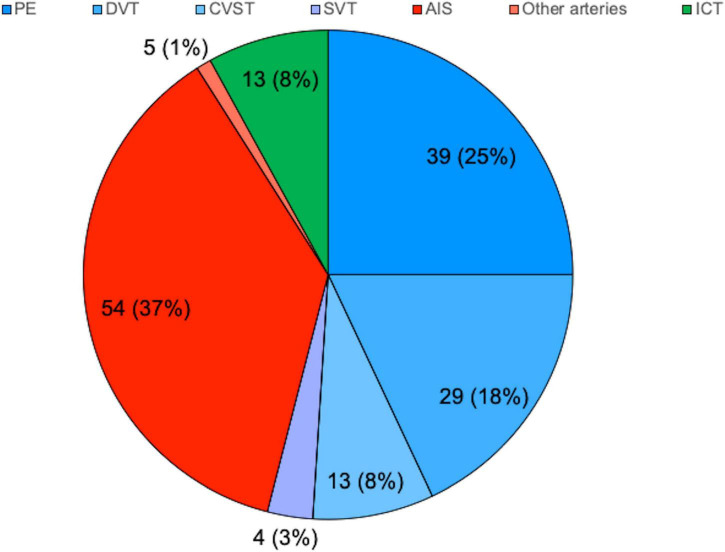
Distribution of thrombotic events by localization. N (%), expressed on the total of 157. AIS, arterial ischemic stroke; CVST, central venous sinus thrombosis; DVT, deep venous thrombosis; ICT, intracardiac thrombosis; PE, pulmonary embolism; SVT, splanchnic venous thrombosis.

**FIGURE 3:**
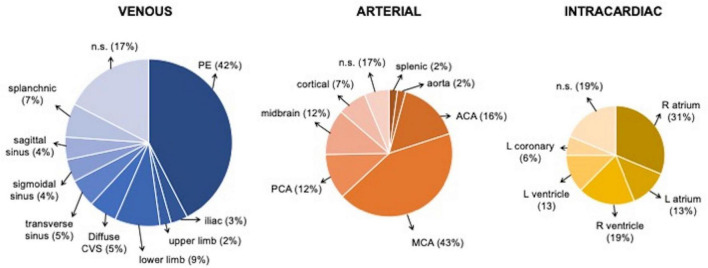
Localization of Venous, arterial, and intracardiac TEs. ACA, anterior cerebral artery; CVS, cerebral venous sinus; L, left, MCA middle cerebral artery; PE, pulmonary embolism; PCA, posterior cerebral artery; R, right.

A total of 62 studies including 138 patients with TEs associated with SARS-CoV-2 infection or MIS-C were included. Most were case reports (53 articles), 3 were case-series including ≥ 3 patients (7, 9, 10), 1 was an observational study (3), and 5 were multicenter cohort studies (5, 11–14). In Supplementary Table 1, (3, 5, 7–66) we reported the clinical and epidemiological characteristics, the sites of thrombosis, the D-dimer values, and data on treatment and outcome of selected cases. Overall, 94 patients had COVID-19 (68%), and 44 had MIS-C (32%). In the whole group, no significant difference in gender was found: 69 children were males (50%), 63 (46%) were females (in 6 cases gender was not specified) (Supplementary Table 1 and Figure 1). The median age was 12 years (IQR 6–15 years), non-etheless age has been assessed only in 101 cases, when it was specifically reported. In 121 children, it was possible to categorize them into 2 age groups: 46/121 (38%) were < 11 years and 75/121 (62%) were ≥ 11 years. Table 1 summarizes the epidemiological data, MIS-C association, risk factors, and outcome of all 138 patients categorized by site of thrombosis. Figure 2 shows the distribution of different types of TEs. The overall number of TEs was 157, as 16 patients developed—simultaneously or subsequently—multiple TEs; VTEs represented the majority (85 events, 54%), followed by ATs (59 events, 38%), and ICT in 13 (8%). Figure 3 displays the localization of thrombosis related to each type of TE: VTE, arterial thrombosis, and ICT.

**TABLE 1:** Epidemiological data, risk factors, MIS-C association, and outcome of the 138 patients with TEs, categorized by site of thrombosis*.

Site of	Patients	Females/Males	Median age	Risk factors**		MIS-C	Death
thrombosis	n	# (%)	(IQR)§	*n* (%)	conditions	n (%)	n (%)
**VENOUS**	**74/138 (54%)**	**42 (57%)/28 (38%)**	**12 y** (8–15)	**47 cases (63%)**	Obesity in 25 cancer in 9 CVL in 18 respiratory in 6	**16/74 (22%)**	**8/74 (11%)**
PE	39 (28%)	25 (64%)/13 (33%)	13 y (11–16)	25 cases (64%)	Obesity in 16 CVL in 3 cancer in 3 asthma in 2 nephrotic syndrome in 2 surgery in 2 diabetes in 2 contraceptives in 2 sickle cell disease in 1	6/39 (15%)	2/39 (5%)
DVT	29 (21%)	16 (55%)/10 (34%)	13 y (10–14)	16 cases (55%)	CVL in 14 obesity in 7 cancer in 5 respiratory in 3 neurologic disorder in 1	10/29 (34%)	5/29 (17%)
CVST	13 (9%)	4 (31%)/9 (69%)	13 y (7–15)	**Cases (38%)**	Infectious in 2 CVL in 1 cancer in 1 obesity in 1 asthma in 1 infection in 1	0	1/13 (8%)
SVT	4 (3%)	2 (50%)/2 (50%)	11 y (8–15)	2 cases (50%)	Obesity in 1 CVL in 1 nephrotic syndrome in 1	0	0
**ARTERIAL**	**59/138** **(43%)**	**17 (29%)/41 (69%)**	**11 y** (5–15)	**28 cases (47%)**	Infections in 7 cardiopathy in 5 ECMO in 5 obesity in 3 CVL in 1	**25/59 (42%)**	**9/59 (15%)**
AIS	54 (39%)	16 (29%)/37 (68%)	10 y (5–15)	27 cases (50%)	Infection in 7 cardiopathy in 5 ECMO in 5 trauma in 3 obesity in 2	20/54 (37%)	**9/54 (17%**)
Other arteries	5 (4%)	1 (20%)/4 (80%)	14 y (6 days–15 y)	1 case (20%)	Obesity in 1	5/5 (100%)	0
**INTRACARDIAC**	13 (9%)	6 (46%)/7 (54%)	11 y (4–12)	**Cases (61%)**	Cancer in 3 CVL in 3 obesity in 2 ECMO in 2 neurologic disorder in 1 surgery in 1	6/13 (46%)	1/13 (8%)
**Total**	138	63 (46%)/69 (50%)	12 y (6–15)	79 cases (57%)	Obesity in 27 (21%) CVL in 23 (18%) Cancer in 13 (10%) Infection in 8 (6%) ECMO in 8 (6%) respiratory in 6 (5%) cardiopathy in 6 (5%) nephrotic syndrome in 3 (2%) neurologic disorders in 3 (2%) surgery in 3 (2%) trauma in 3 (2%) diabetes in 2 (1%) sickle cell diseases in 2 (1%) contraceptives in 2 (1%)	44/138 (32%)	17 (12%)

*16 patients have more than one TEs.

^#^Sex was available in 132 cases.

^§^Age was available in 101 cases.

**Risk factors are available in 130 cases.

These three subgroups had different gender distribution and clinical presentation. Considering the overall number of selected cases (138 patients), we found that the rate of symptomatic TEs was 72% (100/138), the rate of declared asymptomatic ones was 16% (22/138), and in 12% (16/138) the symptoms were not reported.

### Venous thromboembolism

Within the VTEs group, PEs were the most frequent events (39/157 events, 25%), followed by DVT (29 events, 18%), CVST (13 events, 8%), and SVT in 4 (3%). Notably, 10 patients had multiple types of VTEs and four had both venous and arterial thrombosis; among them, a newborn with a thrombus localized both in the right pulmonary artery and into the right ventricle was described (15).

**FIGURE 4:**
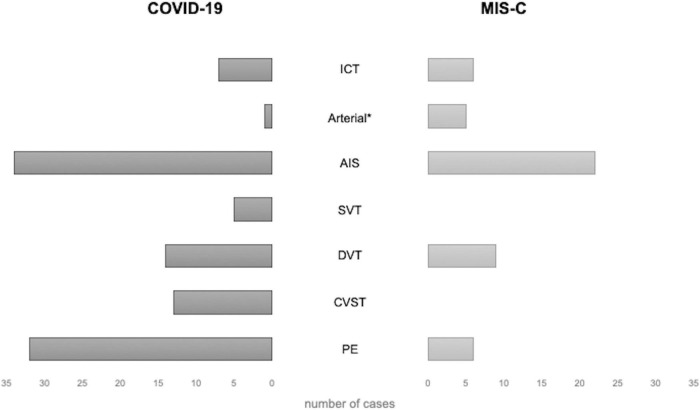
Distribution of thrombotic events by localization in patients with COVID-19 and MIS-C. * excluding AIS. AIS, arterial ischemic stroke; COVID-19, coronavirus disease 2019; CVST, central venous sinus thrombosis; DVT, deep venous thrombosis; ICT, intracardiac thrombosis; MIS-C, multisystem inflammatory syndrome in children; PE, pulmonary embolism; SVT, splanchnic venous thrombosis.

Among the 39 children with PE, 25 (64%) were females, with a median age of 13 years (IQR 11–16 years); 33 cases (85%) had COVID-19, and the remaining 6 had MIS-C (15%) (Figure 4). Their clinical manifestations included severe dyspnea/shortness of breath (12 cases, 31%), cough (4 cases, 10%), hypoxia (4 cases,10%), fever (4 cases, 10%), chest pain (3 cases, 8%), and fatigue (2 cases, 5%); 2 patients (5%) had a cardiac arrest.

DVTs were reported in 29 cases: 16 females [55%, 7 males (24%), and 6 (21%)] unspecified; the median age was 13 years (IQR 10–14). In 10 cases (34%), DVT developed in the context of MIS-C. DVTs were associated with PE in 7 cases, AIS in 2, CSVT and ICT in 1 case each, respectively.

CVSTs were reported in 13 children; 9 (69%) were males, 4 (31%) were females, and the median age was 13 years (IQR 7–15). In 2 patients, CSVT was associated with other TEs (PE and SVT in 1, DVT and PE in another), and in 1 with AIS (16). Clinical presentation included headache in 5 cases (38%), altered consciousness in 5 cases (38%, 1 with coma), hemiparesis/plegia in 2 cases (15%), while photophobia, seizures, right facial palsy, blurred vision, orbital swelling, aseptic meningitis in 1 case (8%) each. All CSVTs occurred in patients with COVID-19; the setting was unspecified in 1 case (5). The most frequent sites of CVST were the transverse sinus (7 cases, 54%), the sigmoid sinus (6 cases, 46%), and the superior sagittal sinus (5 cases, 38%).

SVTs were reported in 4 cases (2 males)—all with COVID-19 (Figure 4)—with a median age of 11 years (IQR 8–15). In 2 cases, SVT was associated with other TEs: PE in one, PE and CVST in the other. The clinical presentation was characterized by abdominal pain and anasarca, in one case each. In a severe case presenting with impaired consciousness, whole body angio-CT with brain CT and angio-MRI were performed, detecting concurrent SVT, CSVT, and PE (17). The sites of SVT were the mesenteric veins in 2 cases, the splenic vein in 1, and the portal vein in 1; the internal jugular, femoral, iliac, and cava were involved altogether in the remaining case.

### Arterial thrombosis

The total number of patients with ATs was 59, including 54 with AIS, 2 with splenic AT (10), 2 with aortic thrombosis in the abdominal (18), and thoracic tract (19), respectively, and 1 with coronary thrombosis (20) (Supplementary Table 1).

The majority of patients with AIS were males (37/54, 68%), with a median age of 10 years (IQR 5–15); 34 children (63%) had COVID-19 and 20 (37%) had MIS-C (Figure 4). The most frequent clinical sign of AIS was hemiparesis/hemiplegia (17 cases, 31%), followed by headache (12, 22%), altered consciousness (8, 15%), cranial nerve paralysis (8, 15%, facial palsy in 7 cases, oculomotor in 1), aphasia (7, 13%), seizures (6, 11%, generalized in 3 cases, focal in 1), dysarthria (4, 7%), mydriasis with anisocoria (4, 7%), nystagmus (3, 5%), and altered gait (3, 5%). Lower limb weakness, upper limb paresis, aseptic meningitis, hypotonia, chorea, hemianopsia, and non-convulsive status epilepticus were reported each in a single case (2%). In 4 patients, AIS was detected in the context of cardiogenic shock, MIS-C, or respiratory distress. The more frequent sites of AIS were middle cerebral artery (MCA) in 41 cases (76%, bilaterally in 5 cases), anterior cerebral artery (ACA) in 14 cases (26%, bilaterally in 2), posterior cerebral artery (PCA) in 11 (20%, bilaterally in 2), and basal ganglia in 5 (15%). Thalamus and brainstem were involved in 2 patients (6%), respectively; the site was unspecified in 5 cases (15%).

### Intracardiac thrombosis

ICT was found in 13 patients (9%): 6 were females (46%), with a median age of 11 years (IQR: 4–12); 7 cases (54%) with COVID-19 and 6 (46%) with MIS-C. The clinical manifestations, reported in 9 cases, included hemodynamic compromise in 7 cases (54%), gastrointestinal symptoms in 3 (23%), and dyspnea with a decreased level of consciousness in 1 (8%). ICT was associated with PE in 3 children (7, 15, 19), and with both AIS and DVT in 1 (21).

### Risk factors

The presence of any risk factors correlated with TEs was reported in 130/138 patients (Supplementary Table 1). Most of them (79 cases, 61%) had at least one predisposing condition. Obesity was the most frequently reported (27/130 cases, 21%), especially in patients with PE (16/39 cases), followed by malignancy (13/130, 10%); other comorbidities included infectious diseases (8 cases, 6%)—2 with tuberculosis, 2 with meningitis, 1 with varicella, 1 with mastoiditis, 1 with brain abscess, and 1 with encephalitis- respiratory diseases (6 cases, 5%), cardiopathy (6 cases, 5%), nephrotic syndrome (3 cases, 2%), neurological disorders (3 cases, 2%), diabetes (2 cases, 1%), and sickle cell diseases (2 cases, 1%). Previous surgery was reported in 3 cases (2%), while the use of drugs (contraceptives) in 2 adolescents (1%). The presence of CVL was documented in 23/130 children (18%), and ECMO was used in 8 patients later developing TEs (6%). The different distribution of the above-mentioned risk factors in the various TEs groups is shown in Table 1.

### D-dimer values

D-dimer values were reported in 86 cases (62%) and were elevated in most patients (75/86, 87%) (Supplementary Table 1). Among them, D-dimers exceeded the upper value of normal (ULN) by at least five times in 52 patients (60%), and by more than 10 times in 27 (31%). The median D-dimer value in reported cases was 3,669 ng/mL (IQR 1,212–11,425). Most patients with PE had markedly elevated D-dimers with median D-dimer value of 6,900 ng/mL. Conversely, D-dimers increased over 10 times the ULN only in 1 case of ICT out of seven reported cases.

### Treatment

Treatment was reported in 97 patients (Supplementary Table 1): 24 (25%) were started on unspecified anticoagulants, 30 children (31%) were started on low molecular weight heparin (LMWH), and 12 (12%) on unfractionated heparin (UFH), while in 13 (13%) unspecified heparin therapy was used. Apixaban was used in 2 patients (13, 22). Twelve cases (12%) were treated with acetylsalicylic acid (ASA) associated with anticoagulation in 9 (23, 24). Thrombolysis was performed in 9 cases (9%): 8 PEs, of which 4 were associated with other TEs, and 1 arterial thrombosis. Eighteen patients (18%) underwent surgery: thrombectomy was performed in 10 cases (1 case with PE and SVT, 1 with PE and DVT, 1 with CVST and AIS, 1 with isolated PE, 5 cases with AIS, and 1 with AT), cardiac surgery was necessary in 4 cases with ICT, 1 patient with DVT had a cava filter positioned, and 1 patient with AIS had an external ventricular drain. Details on surgery were not provided in 2 cases. Several patients received various combinations of drugs for the COVID-19 and/or MIS-C: steroids (17 cases), IVIG (12 cases), hydroxychloroquine (7 cases), and biologic drugs (6 cases). Lopinavir or remdesivir were used in 5 and 3 patients, respectively.

### Outcome

Outcome was reported in 90/138 cases. Most patients (43, 31%) completely recovered, while 17 (12%) improved at discharge or at the last follow-up. Nine patients (6%) had persistent neurological *sequelae* (mostly secondary to AIS), including hemiparesis in 3 children, facial palsy, dysarthria, and aphasia in 2, and weakness in 1. A neonate had abdominal aortic thrombosis requiring leg amputation (18). One patient had a major bleeding event, an adolescent had a premature delivery. In one case, the patient’s outcome was generically reported as “alive.” Seventeen children out of 138 died (mortality rate 12%); most fatalities occurred in patients with ATs (9 cases53%). The death was directly related to AIS in 4 patients (23%); it was due to multiorgan failure in 3/17 cases (18%), cancer in other 3 (18%), cardiac arrest in 2 (12%), and cardiogenic shock in 1 (6%). In 4 cases the death cause was not specified.

## Discussion

### Pathophysiology

**FIGURE 5:**
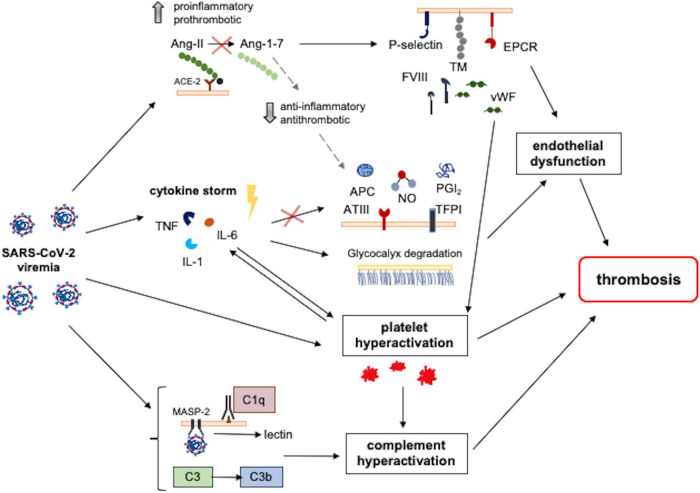
Pathomechanisms involved in SARS-CoV-2-induced thrombosis. Different SARS-CoV-2-induced pathways converge and result in a prothrombotic state. SARS-CoV-2 binding with the ACE2 receptor reduces its activity, increasing Ang2 levels and resulting in increased proinflammatory and prothrombotic signals. These signals mediate endothelial dysfunction and platelet hyperactivation, which is also directly induced by SARS-CoV-2 infection. Contemporary, the hyperinflammatory state induced by SARS-CoV-2 infection contributes to endothelial dysfunction following the reduction of antithrombotic factors and the degradation of glycocalyx. Moreover, SARS-CoV-2 induces complement hyperactivation, resulting in endothelial damage, platelet hyperactivation, and thrombosis. ACE2, angiotensin-converting enzyme 2; Ang, angiotensin; APC, activated protein C; ATIII, antithrombin III; EPCR, endothelial protein C receptor; FVIII, factor VIII; IL-1, interleukin 1; IL-6, interleukin 6; MASP-2, mannose-binding lectin-associated serine protease; NO, nitric oxide; PGI_2_, prostacyclin; TFPI, tissue factor pathway inhibitor; TM, thrombomodulin; TNF, tumor necrosis factor; vWF, von Willebrand factor.

The strong association between COVID-19 and coagulopathy suggests that multiple molecular pathways are involved and dysregulated through the disease progression, contributing to the development of thrombosis. First of all, endothelial dysfunction and barrier disruption lead to immune cell infiltration, and proinflammatory cytokine production, as well as thrombosis (67). In determining such dysfunction, the dysregulation of the renin–angiotensin–aldosterone system (RAAS) and the angiotensin-converting enzyme 2 (ACE2) have a fundamental role. SARS-CoV-2 has higher affinity to ACE2 receptor compared to other coronaviruses. ACE2 is a carboxypeptidase that converts angiotensin II (Ang II) to angiotensin 1–7. The binding between the spike protein (S-protein) of the virus and ACE2 is associated with downregulation of ACE2 activity. In its turn, this will lead to augmentation of Ang II signaling and proinflammatory/pro-thrombotic pathways. On the other hand, the angiotensin 1–7 signaling which mediates anti-thrombotic and anti-inflammatory pathways is diminished. Furthermore, dysregulated RAAS can cause endothelial damage via oxidative stress: the increased reactive oxygen species (generated by Ang II) and reduced nitric oxide (due to low angiotensin 1–7) have detrimental effects on the endothelium. Endothelial dysfunction is also associated with endothelial expression of many prothrombotic molecules and receptors, including P-selectins, angiopoietin-2, and endothelin-1. Moreover, the subendothelial von Willebrand Factor (vWF) is released by damaged endothelium, multimerized by disulfide bonds, and activated by exposing both platelet-binding and collagen-binding domains. Active vWF multimers stick to subendothelial collagen and platelets, activating their aggregation and leading to thrombosis (68). Platelet dysfunction has been, in fact, highly implicated in SARS-CoV-2 infection; activated platelets release various factors for activation of immune responses as well as promotion of the coagulation cascade, including calcium ion and coagulation factors. It has been shown that platelets of COVID-19 patients release significantly larger amounts of cytokines, chemokines, and growth factors upon stimulation than platelets of healthy subjects, and contribute to increased acute phase reactants (fibrinogen, vWF, and factor XII), indicating that platelets are primed to spread proinflammatory and procoagulant activities leading themselves to thrombosis in COVID-19 (67). Thus, innate immunity plays a crucial role as an early defense mechanism against SARS-CoV-2, when uncontrolled initiates the coagulopathic pathways. Several pathways contribute to the dysregulated innate immune response; besides the cytokine storm, also the exaggerated complement activation and excessive neutrophil extracellular traps (NETs), each of which by various mechanisms is involved. The nucleocapsid protein of SARS-CoV-2 binds to the mannose-binding lectin-associated serine protease 2 (MASP-2), expressed on the microvasculature, which leads to complement overactivation. This activation causes the overexpression of endothelial and monocyte tissue factors, enhances platelet activation and endothelial inflammation, further increasing the production of pro-inflammatory cytokines from ECs, and contributing to the cytokine storm. In addition, NETs contain various prothrombotic molecules (i.e., tissue factor, protein disulfide isomerase, factor XII, vWF, and fibrinogen), and release the extracellular DNAs, which directly activate platelets leading to thrombus formation (69). The main pathomechanisms of thrombosis in COVID-19 are summarized in Figure 5. In the pediatric age, COVID-19 and MIS-C seem to have specific effects on coagulation leading to hypercoagulability and thrombogenic state; in particular, complement activation has been hypothesized as a favoring factor in thrombosis development (70). Recent studies in children have pointed to an increase in endothelial dysfunction markers in MIS-C and severe COVID-19, with a rise in soluble C5b-9 (which represents the activated product of the terminal complement cascade) and altered RBC morphology (70, 71). In patients with MIS-C, the high levels of fibrinogen and D-dimers increase the likelihood of a thrombotic state. Furthermore, their prothrombotic state could be explained by the increased clot formation rate and strength. Ankola et al. demonstrated that patients with MIS-C had evidence of hypercoagulability on thromboelastography (TEG); their TEG profiles were consistent with faster clot formation, increased clot strength, and slower fibrinolysis (72). This novel finding has been confirmed by the study of Morparia et al. and provides a potential unique biomarker to distinguish the risk for thrombosis in the pediatric patients (73).

### Epidemiology, clinical manifestations, and management

Severe COVID-19 is frequently burdened by life-threatening thrombotic complications in adults. Conversely, TEs are uncommon in children with COVID-19 and MIS-C. Nevertheless, both these conditions display a proinflammatory perturbation activating the coagulation system, which is emphasized in patients with a complicated clinical course. The earliest case of TE related to COVID-19 in childhood was reported in the first published MIS-C case series, in a 14-year-old boy who died after developing AIS with infarction of right anterior and middle cerebral arteries while on ECMO for acute multiorgan dysfunction, despite anticoagulation with UFH (25). Since then, several relevant original articles on pediatric patients with TEs, including 138 children, have been published. In the first cohort study describing thrombotic complications in 567 children with SARS-CoV-2 infection, the incidence of TEs varied from 0.7% in the global cohort to 1.2% in hospitalized children (11). Although this prevalence is lower than in hospitalized adults with COVID-19, it is slightly higher than that previously described in hospitalized children without COVID-19 (0.13 and 0.55%). A multicenter observational study on MIS-C patients found a TE incidence of 3.8% (9). Similarly, in a US cohort of cases diagnosed with MIS-C, 4 patients (2.2%) developed VTE (74).

In a recent multicenter study, the rate of thrombosis was described in a large cohort of patients < 21 years, hospitalized with SARS-CoV-2 infection or MIS-C, across 7 pediatric hospitals in the United States over 5 months. Overall, 16 patients with VTE, 3 with ICT, and 1 with stroke were described [5]. The rate of TEs in asymptomatic patients with SARS-CoV-2 (0.7%) was similar to that previously reported in hospitalized children (0.58%) (29, suggesting that asymptomatic infection did not increase the thrombotic risk in hospitalized patients). Conversely, in symptomatic patients, the rate of TEs was 2.1% in cases with acute COVID-19, 3.2% in those with MIS-C, and higher in those patients with a severe disease requiring ventilatory support and PICU admission. Data from a multi-institutional cohort study from the Tri-State Pediatric COVID-19 Research Consortium found a similar TE rate (4.3%) considering both patients with COVID-19 and MIS-C, but a lower rate (1.4%) in the patients with MIS-C (75).

Analyzing data from the present review, we found that VTEs are the most frequent thrombotic complications, representing more than half of cases. Among them, PEs and DVTs are the most frequent sometimes with simultaneous localizations. Arterial thrombosis, found in more than one-third of cases, is mainly represented by AIS, while ICTs represent a smaller but relevant group (8%). TEs were mostly found in COVID-19 cases (68% vs. 32%), but this reasonably depends on the relatively low incidence of MIS-C compared with COVID-19. Furthermore, in the setting of MIS-C, ATs occurred more frequently than VTEs. The overall distribution of cases by sex was similar. However, considering different types of thrombosis, we observed that about two third of patients with ATs were males, whereas VTEs occurred more frequently in females (63%). The majority of cases were older than 11 years (62%). The central nervous system was the most commonly affected site, involved in 66 cases (specifically, by AIS in 53 children, CVST in 12, both AIS and CSVT in one), followed by the lungs (PE reported in 39 patients). The heart was affected in 14 cases, mostly with ICT (13 patients), and coronary impairment in 1; the remaining cases had TEs localized at lower/upper limbs (29 cases with DVT) or visceral vessels (4 cases with SVT and 4 AT). Overall, stroke appears to be a rare, but severe complication of COVID-19. Both an International Pediatric Stroke Study survey of 61 international hospitals and a retrospective cohort study at seven US children’s hospitals suggested that the prevalence of stroke among children hospitalized for COVID-19 was < 1% (5, 12). Indeed, COVID-19 has been linked to cardioembolic and arteriopathy stroke and some children had stroke while on ECMO in the setting of MIS-C (25, 26). The mechanism underlying ischemic arterial stroke is not fully clarified; in most articles reporting AIS in children with COVID-19 or MIS-C, this topic is not addressed. Conversely, focal cerebral arteriopathy has been described in several children: in detail, in 4 cases reported by Beslow et al. in the two subsequent surveys (12, 14), in 1 by Gulko et al. (27), and 1 described by Mirzaee et al. (28) The results of these authors suggest focal cerebral arteriopathy as a mechanism of SARS-CoV-2 related AIS; the virus, as well as varicella one, might be a trigger for inflammation and pediatric cerebral arteriopathy leading to stroke. Nevertheless, in the recent survey reported by Beslow et al., vasculitis or arteritis has been proposed to be related to AIS pathogenesis in other 3 cases (14). Last but not least, the presence of an arterial thrombus in some cases has been supported by the fact that these patients underwent thrombectomy (14, 29–31).

About one-third of all the patients were previously healthy, while 57% had a history of one or more comorbidities. ECMO, required for severe cases, represents a medical procedure highly related to TEs development, as well as the presence of CVL, confirming the already well-known association between thrombosis and central venous catheters in children (76). These predisposing conditions are the same reported in children with thrombosis, occurring regardless of SARS-CoV-2 (77). Interestingly, patients with VTs compared with those with ATs had a higher rate of comorbidities. In adults with COVID-19, D-dimer levels are associated with thrombosis and poor outcome (78), while in children their predictive role is quite more controversial. High D-dimers are described in around 20% of children with SARS-CoV-2 infection (79), but their association with thrombosis was found only in children with MIS-C (5). In the present review, most patients with a thrombotic complication had elevated D-dimers, in many cases exceeding the ULN of more than five times. As expected, patients with more severe events such as PE typically had much increased D-dimer values, while in those with ICT the D-dimer elevation was milder. However, these observations are limited by the heterogeneous presentation of data on D-dimer values in the included studies: when possible, we reported the D-dimer value at TE diagnosis, but in some cases, this information was not available, and many studies did not report D-dimer values at all.

Most patients with TEs received anticoagulant treatments. Heparin was used in more than half of the cases: LMWH in 30 cases, followed by UFH (12 cases), often given subsequently in the same patient; rarely other anticoagulants (apixaban), were reported. There is no evidence to support routine prophylactic therapy in patients with COVID-19 or MIS-C (80), and this topic was not within the purposes of our review. Thrombolysis was reserved for those with the most severe TEs, and mostly in patients with PE. Surgery was performed in 19% of cases: 10 underwent thrombectomy, while 4 children with ICT required cardiac surgery. In children with COVID-19, the mortality rate ranged from 12 to 28% in those with TEs (5), compared to around 1% in those without TEs, and most deceased children had fatal comorbidities, mainly cancer. The overall mortality rate from our review was 12%; notably, analyzing the different sites of TEs, it was slightly higher in patients with ATs (15%) compared to those with VTEs (11%). Similarly, in adults with COVID-19, the pooled estimate of mortality with TE is 23% compared with 13% without TE. The relatively low incidence of TEs in the pediatric population requires larger data sets, patient registries, and multi-institutional collaborations, to obtain high-quality evidence to optimally manage this risk in children. Despite a relatively low incidence and frequent benign clinical course of COVID-19 in childhood, its complications such as coagulopathy and TEs, and, in particular, the arterial ones, might represent a leading cause of morbidity and mortality even in pediatric patients.

This study represents a thorough review of the available English literature on a most relevant topic, TE complications in children with COVID-19 or MIS-C, and it provides an extensive understanding of the incidence, clinical manifestations, risk factors, and management of such complications in pediatric age. However, this review has some limitations. First of all, the collection of clinical cases and case series does not allow statistical analysis; therefore, no correlation between TEs and other clinical parameters could be established. Besides, thromboprophylaxis has not been assessed, as this information was not homogeneously reported (or even absent) in the selected articles. The relationship between thromboprophylaxis and TEs was out of the aims of this study and should be further investigated.

## Author contributions

ST and CR conceived the study and prepared the original draft. ST, CR, and FP performed the analysis and prepared tables and figures. DL, GI, MR, and GS critically revised the manuscript. All authors have read and agreed to the final version of the manuscript.
