# Supplementary material for: Thromboembolic complications in children with COVID-19 and MIS-C: A narrative review
Source: Front Pediatr. 2022 Aug 11;10:944743. doi: 10.3389/fped.2022.944743 (PMC9402981; doi:10.3389/fped.2022.944743)
Supplement: Supplementary file 1 [file Table_1.docx]

Supplementary Material

| **eTable 1: epidemiological, clinical features and laboratory findings of 118 children with thromboembolic manifestations affected by COVID-19 or MIS-C** | | | | | | | | | |
| --- | --- | --- | --- | --- | --- | --- | --- | --- | --- |
| **Author** | **Sex/age** | **Risk factors** | **Type of thrombosis** | **Clinical features** | **Site** | **D-dimer**  ng/mL | **MIS-C** | **Therapy** | **Outcome** |
| Aghaei Moghadam (32) | M/18m | ALL | Intracardiac | Fever,rash, cough diarrhea, vomiting | R SVC in R atrium | 4000 | No | Enoxaparin | Recovery |
| Aguilera-Alonso (11) | F/4y | systemic juvenile idiopathic arthritis, CVL | DVT | NS | R iliac vein | 5,953 | No | LMWH | Recovery |
|  | F/12y | ALL, obesity,  CVL | DVT | NS | R upper limb | 232 | No | LMWH | Recovery |
|  | F/13y | None | DVT | NS | L common and superficial femoral vein | 1,194 | No | LMWH  cava filter | Recovery |
|  | F/13y | None | DVT  PE, CVST | NS | Transverse sinuses jugular  femoral vein | 35,420 | No | Continuous IV UFH  LMWH | Recovery |
| Alfoudri(33) | F/8y | Obesity | PE | Fever cough, respiratory distress, hypoxia |  | >20,000 | No | ECMO  Tocilizumab  Thrombolysis | Recovery |
| Amonkar(18) | M/6d | None | Arterial thrombosis | R leg gangrene | Abdominal aorta | 4,110 | Yes | UFH  thrombolysis  corticosteroids  embolectomy | Recovery after amputation |
| Anastas(34) | F/15y | Asthma | PE | Cardiac arrest | Massive bilateral | >20,000 | Yes | Thrombolysis  UFH | Neurologic *sequelae* |
| Anvekar(35) | M/12y | None | CVST | Fever, headache  Altered mental status | sigmoid, lateral, and jugular venous sinuses | 1,280 | No | LMWH | Improved |
| Appavu(29) | F/8y | None | AIS | R hemiplegia, language impairment | Bilateral MCA | 1,830 | No | Thrombectomy  anticoagulant  ASA  corticosteroids | Partial recovery: aphasia & dysarthria |
|  | M/16y | None | AIS | R hemiparesis  Aphasia | L MCA | 6,060 | No | UFH  LMWH | Partial recovery: dysarthria, aphasia, R FP, R arm weakness |
| Asif (36) | M/18y | None | CVST | Headache  Photophobia  Fever | Sigmoid, transverse sinuses  straight and superior sagittal sinuses | NS | No | LMWH | Improved |
| Beslow 2021 (12) | F/4d | None | AIS | Encephalopathy, jitteriness, nystagmus, severe hypotonia | Thalamo-capsular | NS | No | NS | NS |
|  | F/28m | Varicella | AIS | Chorea | L basalganglia, L MCA | NS | No | NS | NS |
|  | F/32m | None | AIS | Fever, gait disturbances, oculomotor abnormalities | Midbrain | NS | No | NS | NS |
|  | M/10y | None | AIS | Fever, respiratory distress, focal seizures | Bilateral PCA | NS | No | NS | NS |
|  | M/10y | Sickle cell disease | AIS | Fever, MIS-C, chest pain, back pain, abdominal distension | R MCA  small cerebellar infarcts | NS | Yes | NS | NS |
|  | M/14y | ECMO | AIS | Fever, headache, MIS-C | R MCA, R ACA | NS | Yes | NS | NS |
|  | M/16y | Lemierre syndrome Meningitis | AIS | Fever, headache, ear effusion, otalgia | L MCA, L ACA  R ACA | NS | No | NS | NS |
|  | M/9y | Mastoiditis | CVST | Cough | R transverse sinus  R sigmoid sinus | NS | No | NS | NS |
| Beslow 2022 (14) | M/2y | Wiskott Aldrich syndrome, bone marrow transplant, recent brain abscess, hypertension | AIS | Altered mental status, seizures | R MCA and PCA | 4,572 | Yes | NS | NS |
|  | M/6y | None | AIS | R hemiparesis, headache | L MCA | 141.9 | No | NS | NS |
|  | M/8y | None | AIS | COVID19 symptoms and MIS-C | L MCA | 1,310 | Yes | NS | NS |
|  | F/8y | None | AIS | R hemiparesis, aphasia | Bilateral MCA | 1,830 | No | Trombectomy | NS |
|  | M/16y | None | AIS | R hemiparesis and aphasia | L MCA | 6,060 | No | NS | NS |
|  | F/16y | None | AIS | L FP, L hemiparesis, headache | R ACA and MCA | 280 | No | NS | NS |
|  | M/1y | Tetralogy of Fallot, arrhythmia | AIS | NS | L ACA, R MCA | NS | No | NS | NS |
|  | F/1y | Septic shock, abdominal compartment syndrome | AIS | NS | R MCA | 2,122 | No | NS | NS |
|  | M/3y | Cervicocephalic dissection | AIS | R hemiparesis | L MCA, L ICA | 2,682 | No | NS | NS |
|  | M/4y | Hydrocephalus, dehydratation, Plasminogen Activator Inhibitor-1 homozygous | AIS | Headache, vomiting, abdominal pain, poor oral intake, refusal to ambulate | L ACA, R ACA, R PCA | 490 | No | NS | NS |
|  | F/12y | Trisomy 21, moyamoya | AIS | Difficulty ambulating, R FP | L MCA | 2,020 | No | Thrombectomy | NS |
|  | M/13y | Possible rheumatic disease | AIS | Headache | L MCA | 840 | No | Trombectomy | NS |
|  | M/14y | L ventricle myxoma, homozygous MTHFR mutation | AIS | Nystagmus, diplopia, restricted up and downgaze, anisocoria | L PCA | 419 | No | NS | NS |
|  | F/14y | None | AIS | Neurological deficits | Anterior spinal artery | 440 | No | NS | NS |
|  | F/15y | Trisomy 21, mitral regurgitation, moyamoya | AIS | Generalized tonic clonic seizure | R ACA, R MCA | 1230 | No | NS | NS |
|  | M/15y | Multiple dissections after trauma, low protein C, low protein S | AIS | Headache, neurological deficits | R MCA, R PCA | 1230 | No | NS | NS |
|  | M/16y | Takayasu vasculitis | AIS | Neurological deficits | R ACA, MCA, PCA | 171 | No | NS | NS |
|  | M/17y | Hodgkin’s lymphoma, Cryptococcus, meningitis, presumed vasculitis | AIS | Headache, generalized malaise | L MCA, L PCA, R ACA, R MCA, cerebellum | NS | No | NS | NS |
|  | M/17y | Acute anemia after accident | AIS | Neurological deficits | L MCA, L PCA, R ACA, R MCA, R PCA | NS | No | NS | NS |
|  | F/2y | Moyamoya, pleural arteriovenous malformation, porto-cava malformation, human Herpesvirus 6 + in cerebrospinal fluid | AIS | Neurological deficits | L MCA | NS | No | NS | NS |
|  | M/14y | Patent forameovale | AIS | Neurological deficits | L MCA | NS | No | NS | NS |
|  | M/16y | Head and neck trauma | AIS | NS | R PCA | 109 | No | NS | NS |
| Bigdelian 2021 (7) | F/11y | None | Intracardiac | Fever, tachycardia | L atrium | 490 | No | HCQ, lopinavir;  Cardiac surgery | Recovery |
|  | F/7y | None | Intracardiac | Fever, tachypnea, rash, hypotension, vomit | L atrium | 530 | Yes | HCQ, lopinavir, IVIG, corticosteroids, LMWH;  Cardiac surgery | Recovery |
|  | F/8y | Orthopedic surgery | PE  Intracardiac | Fever, tachypnea, tachycardia | L ventricle, right cardiac chambers, pulmonary artery | 2,900 | No | HCQ, lopinavir, IVIG, corticosteroids, LMWH;  Cardiac surgery | Recovery |
| Bigdelian 2020 (37) | M/11y | Seizure | Intracardiac | Fever, dyspnea  decreased consciousness | Tricuspid valve  R atrium and ventricle | NS | No | HCQ, lopinavir;  Cardiac surgery | Recovery |
| Bin Ali (38) | F/12y | None | PE | Loin pain, dry cough, dyspnea  Chest pain | extensive bilateral  pulmonary embolisms | 4,590,000 | No | LMWH  HCQ  Azathioprine | Improved |
| Blazkova(39) | M/2m | None | CVST | R-sided facial  muscle spasms and L upper limb spasm, seizures, fever | Transverse, sigmoid sinuses, confluenssinuum, and sagittal superior sinus. | NS | No | LMWH | Recovery |
| Blumfield(40) | F/6y | NS | PE | NS | L upper lobe artery | NS | Yes | NS | Recovery |
|  | NS/NS | NS | PE | NS | Segmental | NS | Yes | NS | Recovery |
| Caro-Dominguez (41) | M/16y | NS | PE  DVT | R leg pain  Tachycardia | Bilateral segmental arteries of lower lobes | NS | No | NS | NS |
| Chang (21) | F/15y | None | AIS | Aphasia  R hemiparesis | L MCA  L caudate, insula, frontal operculum | NS | Yes | IVIG  corticosteroids  LMWH | subtle R hemiparesis then recovery |
|  | F/16y | None | L ICA AIS  R limb DVT  Intracardiac | Hypotension  Aphasia, Lethargy  R hemiparesis | L insula, caudate, frontal and temporal lobes  R limb | NS | Yes | IVIG  corticosteroids,  LMWH, ASA | Mild aphasia |
| Chima(13) | 6 F  2 M  16.5y median | 2 obesity  1 contraceptive  1 surgery  1 cancer | 5 PE  3 PE+ DVT | NS | NS | NS | No | 7 heparin  1 Apixaban  1 ASA  1 IVIG+ corticosteroids | Recovery |
| Cristoforo (42) | M/11y | Obesity  Nephrotic syndrome | PE | shortness of breath  fatigue  lower limb swelling | Saddle  pulmonary embolisms | 74,443 | No | Heparin  Thrombolysis | Recovery |
| Cursi (8) | M/9d | None | CVST | Fever  Respiratory failure  Enterorrhagia | Medullary vein thrombosis | 1,770 | No | Antibiotics  LMWH  Remdesivir | Recovery |
| Dakay(43) | M/17y | Obesity | CVST | Headache, emesis  Blurred vision | L transverse and sigmoid superior sagittal sinuses | 1,130 | No | LMWH | Improved |
| Davies (9) | 3 cases | NS | DVT | NS | NS | NS | Yes | NS | NS |
| Essajee(23) | F/31m | TB | CVST | L-side weakness  Lethargy | Superior  sagittal transverse sinuses | 14,800 | No | ASA | L hemiparesis |
| Fraser (44) | M/6m | TB | AIS | Cough, labored breathing, reduced oral intake | punctate L thalamic stroke, proximal R MCA and PCA | NS | No |  | Died (multiorgan failure) |
| Gulko(27) | F/13y | None | AIS | Headache, speech difficulty, R limbs weakness | LMCA | NS | Yes | NS | Improved |
| Hameed (10) | M/15y | NS | Splenic artery | NS | Splenic infarct | NS | Yes | IVIG, corticosteroids, biologics  anticoagulation NS | Recovery |
|  | NS/NS | NS | Splenic artery | NS | Splenic infarct | NS | Yes |  | Recovery |
|  | NS/NS | ECMO | AIS | Altered neurologic status | ACA, MCA | NS | Yes |  | Died (NS cause) |
| Hodes (45) | M  F  19.5 y mean | Obesity, hypertension | PE | NS | Segmental | 700 | No | NS | Recovery |
|  |  | Obesity,  diabetes  asthma | PE | NS | Segmental, bilateral | 1,100 | No | NS | Recovery |
| Hussain (46) | M/14y | None | DVT  PE | Limb swelling  Dyspnea, chest pain, cough, fever | Left femoral vein | NS | No | LMWH followed by warfarin  ASA  HCQ  Corticosteroids | Recovery |
| Hussein (47) | M/20y | Nephrotic syndrome  Obesity | SVT  PE | Aneusmia, ageusia, hypotension, anasarca | portal, splenic, mesenteric and R hepatic veins | NS | No | Thrombectomy  Thrombolysis  Heparin | Improved |
| Ippolito Bastidas(17) | F/13y | None | CVST  PE  SVT | Headache  impaired consciousness | R intracerebral hemorrhage  Transverse, R sigmoid,  superior sagittal sinuses  internal jugular, femoral, iliac, pulmonary, cava | 33,960 | No | UFH  lopinavir-ritonavir HCQ  antibiotics | Recovery after 24 days of admission |
| Kangin(48) | F/7y10m | None | AIS | Generalized seizures  listless, aphasia | MCA, ACA  L frontal and parietal lobes | 3,669 | No | NS | Died (AIS) |
| Kaushik (26) | M/5y | None | AIS | Shock cardiogenic | R ACA and MCA | 18,300 | Yes | Heparin  Tocilizumab  ECMO | Died (NS cause) |
| Kenchappa(49) | F/10y | None | SVT | Abdominal pain,  Nausea | mesenteric vessels | 1,496 | No | LMWH  Surgery | Improved |
| Keskin(50) | M/9y | None | AIS  DVT | Confusion  L-sided FP hemiparesis  Swollen R arm | NS  R median antebrachial vein | NS | Yes | IVIG  corticosteroids  LMWH, ASA | L central FP |
| Khosravi(51) | M/10y | None | AIS | Headache  VII nerve paralysis  Tonic movements  Consciousness loss | R MCA  R putamen, globus pallidus, posterior insula | Normal | No | LMWH  ASA | Improved |
| Kihira(52) | M/5y | None | AIS | Fever, cough  Hypotension  Cardiogenic shock  R pupil dilated- unreactive | MCA | 12,000 | Yes | ECMO | Died (cardiogenic shock) |
| Kotula(53) | F/15y | Obesity  Appendectomy | PE | Syncope  Shortness breath  Hypertension  Cardiac arrest | pulmonary arteries and superior vena cava | >20,000 | Yes | Thrombolysis | Improved |
| Lindan(54) | F/15y | None | CVST | fever, confusion headache |  | NS | No |  | Recovery |
| Martinelli(55) | F/17y | Obesity  Pregnancy | PE | Acute respiratory failure | R superior lobe | 16,400 | No | LMWH | Premature delivery |
| Minen(56) | M/14y | Obesity  ECMO | AIS | Fixed anisocoria | R ACA and MCA | 6,440 | Yes | UFH | Died (NS cause) |
|  | F/12y | ECMO | Intracardiac | NS (inside MIS-C vasoplegic shock) | R atrium | 69,400 | Yes | UFH  ASA  corticosteroids  biologic | Recovery |
| Mirzaee(28) | M/12y | None | AIS | Generalized seizures  R hemiparesis  Dysarthria | L MCA  L basal ganglia and insula | NS | No | NS | Hemiparesis |
| Mitchell (3) | M/2m | CVL | SVT | NS | Portal vein | 10,090 | No | LMWH | Recovery |
|  | F/4y | CVL | DVT | NS | R internal jugular vein | 10,850 | No | LMWH | Recovery |
|  | F/8y | None | PE | NS | Bilateral PE | 13,480 | No | LMWH | Recovery |
|  | M/11y | Genetic syndrome, CVL | PE | NS | - | 3,810 | No | LMWH | Recovery |
|  | F/14y | Genetic syndrome, CVL | DVT | NS | R lower limb | 16,190 | No | LMWH | Died (NS cause) |
|  | M/19y | Obesity, CVL | PE | NS | - | 6,260 | No | LMWH | Recovery |
|  | M/21y | None | PE | NS | - | 4,950 | No | LMWH | Recovery |
| Odievre(57) | F/16y | Sickle cell disease | PE | Fever, cough, distress,  chest pain, ACS | Bilateral pulmonary embolism | 23,611 | No | Anticoagulant  Tocilizumab | Improved |
| Oualha(16) | M/16y | None | CVST, AIS | Aseptic meningitis, hemiparesis, coma | MCA | NS | No | Surgical drainage  anticoagulant | Died (AIS) |
| Ouarradi(58) | F/14y | Obesity | PE | Severe dyspnea | Massive bilateral | 3,520 | No | UFH | Recovery |
| Pabst (30) | M/NS | None | AIS | L hemiplegia, L facial droop, homonymous hemianopsia, dysarthria | R MCA | >ULN | Yes | Endovascular thrombectomy | Recovery |
| Panjabi (22) | F/15y | Obesity  contraceptives | PE | Chest pain  dyspnea | Bilateral pulmonary arteries | NS | No | UFH followed by LMWH  corticosteroids | Improved |
|  | F/16y | Obesity  diabetes | PE | Fatigue, cough  shortness breath | Pulmonary saddle, distal pulmonary branches | 6,900 | No | Embolectomy, Continuous IV UFH Apixaban | Improved |
| Persson (20) | F/15y | None | Coronaries | Dyspnea,  Chest pain | L coronary artery | 920 | Yes | ASA , IVIG  heparin  eptifibatide  clopidogrel  remdesivir | improved |
| Riphagen(25) | M/14y | obesity | AIS | Fever headache | R MCA and ACA | 13,400 | Yes | Dopamine, IVIG  noradrenaline  corticosteroids | Died (AIS) |
| Scala (31) | M/11y | None | AIS | L- hemiplegia,  dysarthria,  lateral nystagmus | R MCA | 3,188 | No | Hemicraniectomy  External ventricular drain  heparin | severe hemiparesis |
| Schultze-Schiappacasse(15) | M/27d | None | PE  Intracardiac | Hypotensive shock  Dehydration  Cyanosis, hypoxia | R ventricle  R pulmonary artery | NS | No | Amine  Thrombolysis  LMWH | Improved |
| Schroder (59) | M/17y | None | Intracardiac | Fever, emesis, hemodynamic shock | L ventricular apex | 3,800 | Yes | LMWH, IVIG  corticosteroids  biologic, ASA | recovery |
| Shen (24) | M/17m | None | AIS | R arm and  leg weakness | L pontine | Normal | No | ASA | Mild weakness |
| Shobhavat(60) | F/12y | NS | AIS | Upper limb monoparesis, confusion | L posterior periventricular white matter | NS | Yes | NS | Alive |
| Schupper(61) | M/5y | ECMO | AIS | R mydriasis | R MCA | NS | Yes | UFH | Died (AIS) |
|  | M/2m | Tracheostomy  ECMO | AIS | status epilepticus | Bilateral MCA and PCA | NS | yes | NS | NS |
| Thomas (62) | M/6y | None | AIS | Acute hemiplegia  Aphasia  VII nerve paralysis | NS | 5,061 | Yes | LMWH  IVIG, corticosteroids,  ASA | Recovery |
| Tiwari (63) | F/9y | None | AIS | Headache  R hemiplegia  R VII nerve palsy | R MCA  intracranialinternalcarotids | 3,570 | Yes | IVIG  corticosteroids  Remdesivir  LMWH | Improved |
| Turbin(64) | M/15y | Asthma | ophthalmic thrombosis  CVST | Orbital swelling  Fever | cavernous sinus and dural venous sinuses  Superior ophthalmic vein | NS | No | Surgery  LMWH | Improved |
| Whitworth (5) | F/16-18y | Cancer, CVL obesity | PE | 13 symptomatic  7 asymptomatic | NS | >5xULN in 14 pts  >ULN in 2 pts  not done in 2 pts  NS in 2 pts | No | Anticoagulant | Died (cardiac arrest) |
|  | M/16-18y | congenital heart disease, CVL  neurologic disorder  respiratory disorder | DVT |  | NS |  | No |  | Died (cardiac arrest) |
|  | M/18-21y | Cancer, CVL | PE |  | NS |  | No |  | Died (multiorgan failure) |
|  | M/16-18y | Obesity | DVT |  | NS |  | No |  | NS |
|  | F/<1y | Cancer, CVL | Intracardiac |  | NS |  | No |  | Died (cancer) |
|  | F/10-12y | Obesity | PE |  | NS |  | No |  | NS |
|  | M/12-14y | Cancer | CVST |  | NS |  | No |  | Major bleeding |
|  | F/14-16y | Cancer, obesity, CVL | DVT |  | NS |  | No |  | NS |
|  | M/14-16y | Cancer, CVL | Intracardiac |  | NS |  | No |  | NS |
|  | M/16-18y | Neurologic disorder, CVL | AIS |  | NS |  | Yes |  | NS |
|  | F/12-14y | Respiratory disorder, CVL | DVT |  | NS |  | Yes |  | NS |
|  | F/14-16y | CVL, ECMO | DVT |  | NS |  | Yes |  | NS |
|  | M/16-18y | CVL | DVT |  | NS |  | Yes |  | NS |
|  | M/16-18y | Obesity, CVL, ECMO | Intracardiac |  | NS |  | Yes |  | NS |
|  | F/16-18y | Obesity, CVL | DVT |  | NS |  | Yes |  | NS |
|  | F/14-16y | Cancer, obesity, respiratory disorder, CVL | DVT |  | NS |  | Yes |  | Died (multiorgan failure) |
|  | M/18-21y | Cancer, obesity, CVL | DVT |  | NS |  | Yes |  | NS |
|  | F/16-18y | Obesity, CVL | DVT |  | NS |  | Yes |  | NS |
|  | F/16-18y | Cancer | DVT |  | NS |  | No |  | Died (cancer) |
|  | F/<1y | acute liver failure, CVL | DVT |  | NS |  | No |  | Died (cancer) |
| Winant (65) | F/6y | NS | PE | Hypoxic respiratory failure | L upper lobe segmental artery | NS | Yes | NS | NS |
| Woods (19) | M/12y | Obesity | PE  thoracic aorta  Intracardiac | Fever, pain chest  Dyspnea | small R atrial (RA)  thoracic aorta  bilateral pulmonary arteries | 3,954 | Yes | UFH  Thrombolysis | Recovery |
| Visveswaran(66) | F/12y | None | DVT  PE | swollen, painful, cyanotic L limb  hypotension  RV hypokinesis | iliacvein, posteriortibialvein  R lung | 1,953 | No | Thrombectomy  ECMO  Thrombolysis  corticosteroids  Heparin | Improved |

ACA: anterior cerebral artery; ACS: acute chest syndrome, AIS: arterial ischemic stroke, ALL acute lymphoblastic leukemia, ASA: acetylsalicylic acid, CVL: central venous line, CVST: central venous sinus thrombosis, d: days, DVT: deep venous thrombosis, ECMO: extracorporeal membrane oxygenation, F: female; FP: facial palsy; HCQ: Hydroxychloroquine; ICA: internal carotid artery; IV: intravenous, IVIG: intravenous immunoglobulin, L: left, m: months, LMWH: low molecular weight heparin, M: male, MCA: middle cerebral artery, MIS-C: multisystem inflammatory syndrome in children, NS: Not specified, PCA: posterior cerebral artery, PE: pulmonary embolism; R: right, SVT: splanchnic venous thrombosis, TB: tuberculosis, UFH unfractionated heparin, ULN: upper level of normal, y: year
